# Point estimation following a two-stage group sequential trial

**DOI:** 10.1177/09622802221137745

**Published:** 2022-11-16

**Authors:** Michael J Grayling, James MS Wason

**Affiliations:** 5994 Newcastle University, Newcastle upon Tyne, UK

**Keywords:** Adaptive design, bias, early stopping, interim analysis, mean squared error, uniform minimum variance unbiased estimator

## Abstract

Repeated testing in a group sequential trial can result in bias in the maximum likelihood estimate of the unknown parameter of interest. Many authors have therefore proposed adjusted point estimation procedures, which attempt to reduce such bias. Here, we describe nine possible point estimators within a common general framework for a two-stage group sequential trial. We then contrast their performance in five example trial settings, examining their conditional and marginal biases and residual mean square error. By focusing on the case of a trial with a single interim analysis, additional new results aiding the determination of the estimators are given. Our findings demonstrate that the uniform minimum variance unbiased estimator, whilst being marginally unbiased, often has large conditional bias and residual mean square error. If one is concerned solely about inference on progression to the second trial stage, the conditional uniform minimum variance unbiased estimator may be preferred. Two estimators, termed mean adjusted estimators, which attempt to reduce the marginal bias, arguably perform best in terms of the marginal residual mean square error. In all, one should choose an estimator accounting for its conditional and marginal biases and residual mean square error; the most suitable estimator will depend on relative desires to minimise each of these factors. If one cares solely about the conditional and marginal biases, the conditional maximum likelihood estimate may be preferred provided lower and upper stopping boundaries are included. If the conditional and marginal residual mean square error are also of concern, two mean adjusted estimators perform well.

## 1. Introduction

A desire to reduce the number of patients required in a clinical trial has seen a large number of trialists utilise a group sequential study design. In this approach, interim analyses after certain landmark amounts of data have been collected afford the possibility for a trial to terminate early. Approaches to design for fixed sample studies are not appropriate with repeated data analysis, due to inflation of error rates. Therefore, a large amount of methodology has been developed to facilitate attaining desired type I and type II error rates in a group sequential trial. For an overview of this methodology, see Jennison and Turnbull^1^ or Whitehead.^2^

In addition, following a group sequential trial, special inferential techniques are required. In the case of confidence intervals, this is to ensure the desired coverage is attained. For p-values, it is to guarantee that they are consistent with the decision about the null hypothesis. Here, our focus is on point estimation, where specially developed methods are required because the standard maximum likelihood estimator (MLE) is no longer either unbiased or minimum variance.

Numerous authors have now proposed point estimation procedures for after a group sequential trial. Often, these have sought to reduce the bias in the estimate; an important aim given that the magnitude of a treatment effect is always of principal interest in a clinical trial. However, there are several important factors to consider when choosing a preferred estimator, including also the residual mean squared error (RMSE), and the division of the bias and RMSE into their marginal and conditional (on stage of termination) values.

Several authors have also sought to compare estimators.^3–6^ However, each of these works has compared a limited number of estimators, and has been set within the context of a specific study setting (e.g., Shimura^6^ consider survival data). Recently, Robertson et al.^7^ provided an extended discussion of adjusted point estimation following adaptive design. Along with describing available methodologies, they provided guidance for researchers on best practice, and reviewed current use in published trials. They found, like Zhang et al.,^8^ that to date few studies have computed an adjusted estimate. Shimura et al.^6^ previously argued this is because of a lack of available comparisons of estimators and lack of software for their computation. Robertson et al.^7^ also noted the potential importance of conducting an extensive comparison of estimators to choose the preferred approach for a given trial. The purpose of this paper is therefore to describe a large number of estimators, and historical results pertaining to them, within a common notation. Through this, we make the principal evaluations that are required to compare estimators clear. We then examine the performance of these estimators in terms of their biases and RMSE through several informative examples. By focusing on a certain type of two-stage trial, we also derive a number of small new results that simplify the determination of several estimators in practice. Furthermore, we provide code for implementation such that modification for alternative settings can be readily achieved. We proceed by first describing the exact design setting considered.

### 2. Methods

#### 2.1. Design setting

We consider point estimation following a two-stage group sequential trial that bases decisions on the standardised test statistics $\left\{Z_{1},Z_{2}\right\}$, which follow the canonical joint distribution with information levels $\left\{I_{1},I_{2}\right\}$ for the parameter θ, as defined by Jennison and Turnbull.^1^ That is

-
$\left(Z_{1},Z_{2}\right)$ is bivariate normal,
-
$E(Z_{k})=\theta \sqrt{I_{k}},\;k=1,2$, and
-
$Cov(Z_{k_{1}},Z_{k_{2}})=\sqrt{I_{k_{1}}/I_{k_{2}}}$, $1\leq k_{1}\leq k_{2}\leq 2$.

At least approximately, this joint distribution holds for an extremely wide variety of trial designs and study endpoint types. We assume $H_{0}\;:\;\theta =0$, though this will be rarely used. We focus also on the case where the trial continues to stage 2 if $z_{1}\in (l,u)$. We make these restrictions for several reasons. Firstly, a large proportion of all group sequential trials include only a single interim analysis; see, for example, the review by Stevely et al.^9^ who found two-stage designs to be the most common, accounting for 44.4% (28/63) of included trials for which the number of interims was determinable. Secondly, assuming a continuous continuation region of the form $z_{1}\in (l,u)$ enables the arguably preferable stage-wise test statistic ordering to be employed when determining a median unbiased estimate (MUE) later. Finally, by making these restrictions we are able to develop new explicit solutions for certain point estimators that will aid their determination in practice. No additional assumptions are required for our specification of the point estimators that follow; additional assumptions, as required by specific study setting examples, are given in the Results as needed.

We let K=1,2 denote the number of analyses performed when the trial is terminated and $Z=Z_{K}$ be the corresponding terminal standardised test statistic. Then, the pair of statistics (K,Z) is known to be a sufficient statistic for θ.^3,10,11^ We will make repeated use of the density of (K,Z) given θ, f(k,z|θ). Armitage et al.^12^ described a generic recursive approach to calculating f(k,z|θ). In the Supplemental Materials, we demonstrate that in our two-stage case, it can be written as

(1) $$\begin{array}{rl} \;f(1,z|\theta ) & =\left\{ \begin{array}{cc} \phi (z,\theta \sqrt{I_{1}},1) & \;:\;z\notin (l,u)\\ 0 & \;:\;z\in (l,u) \end{array}\right.\\ f(2,z|\theta ) & =\sqrt{I_{2}}\int_{l}^{u}\phi (z_{1},\theta \sqrt{I_{1}},1)\phi \{z\sqrt{I_{2}}-z_{1}\sqrt{I_{1}},\theta (I_{2}-I_{1}),I_{2}-I_{1}\}\;dz_{1}\\ & =\frac{e^{-(z-\theta \sqrt{I_{2}}{)}^{2}/2}}{\sqrt{2\pi }}[\Phi \{u,z\sqrt{I_{1}/I_{2}},(I_{2}-I_{1})/I_{2}\}\\ & \;-\Phi \{l,z\sqrt{I_{1}/I_{2}},(I_{2}-I_{1})/I_{2}\}] \end{array}$$

where $\phi (x,\mu ,\sigma^{2})$ and $\Phi (x,\mu ,\sigma^{2})$ are respectively the probability density and cumulative distribution functions of a $N(\mu ,\sigma^{2})$ random variable evaluated at x. This final form for f(2,z|θ), which removes the need for integration, is particularly helpful for reducing computational run time when considering the performance of possible estimators in depth; it facilitates the large number of comparisons performed later and the code made available.

Note that f(k,z|θ) can be used to compute the probability of stopping at stage k, which we denote by s(k|θ), with

$$s(k|\theta )=\int_{-\infty }^{\infty }f(k,z|\theta )dz$$

We will then also utilise $f_{\text{cond}}(k,z|\theta )=f(k,z|\theta )/s(k|\theta )$, the conditional density of Z given K=k. Note that in the two-stage case, we must have that s(1|θ)=1−s(2|θ).

#### 2.2. Point estimation

We compare the performance of nine-point estimators for θ, which we denote by $\hat{\theta }=\hat{\theta }(K,Z)$, omitting explicit dependence on (K,Z). Estimates, as defined by particular (k,z), will always be denoted by $\hat{\theta }(k,z)$. Before we specify these estimators, we first define the measures used to compare them. We compute the bias and RMSE of point estimator $\hat{\theta }$, conditional on K=k, as

$$\begin{array}{rl} \mathrm{Bias}(\hat{\theta }|\theta ,k) & =E(\hat{\theta }|\theta ,k)-\theta \\ \mathrm{RMSE}(\hat{\theta }|\theta ,k) & =\sqrt{Var(\hat{\theta }|\theta ,k)+\mathrm{Bias}(\hat{\theta }|\theta ,k{)}^{2}}\\ E({\hat{\theta }}^{x}|\theta ,k) & =\int_{-\infty }^{\infty }\hat{\theta }(k,z{)}^{x}f_{\text{cond}}(k,z|\theta )dz,\;x=1,2\\ Var(\hat{\theta }|\theta ,k) & =E({\hat{\theta }}^{2}|\theta ,k)-E(\hat{\theta }|\theta ,k{)}^{2} \end{array}$$

From these, we can also compute the marginal bias and RMSE of $\hat{\theta }$ as

$$\begin{array}{rl} \mathrm{Bias}(\hat{\theta }|\theta ) & =\sum_{k=1}^{2}s(k|\theta )\mathrm{Bias}(\hat{\theta }|\theta ,k)\\ \mathrm{RMSE}(\hat{\theta }|\theta ) & =\sum_{k=1}^{2}s(k|\theta )\mathrm{RMSE}(\hat{\theta }|\theta ,k) \end{array}$$

That is, the marginal values are simply a weighted sum of the conditional values, with weights given by the probability of the trial stopping at each stage.^13^ Note that we then say $\hat{\theta }$ is unbiased if $\mathrm{Bias}(\hat{\theta }|\theta )=0$ for all θ. Liu et al.^13^ defined $\hat{\theta }$ to be conditionally unbiased if $\mathrm{Bias}(\hat{\theta }|\theta ,k)=0$ for all θ and k. While the latter implies the former, vice versa is not true, and hence being conditionally unbiased is a stronger requirement. Previous examinations of possible point estimators have focused on the fact that low marginal bias may not result in low conditional bias.^13–15^ Thus consideration of both conditional and marginal performance is important.

#### 2.2.1. Maximum likelihood estimator

The MLE of θ is given by ${\hat{\theta }}_{\text{M}LE}(k,z)=z/\sqrt{I_{k}}$; see Chang^16^ for details. As has been remarked on extensively, ${\hat{\theta }}_{\text{M}LE}$ is a biased estimator for θ. Typically, the MLE overestimates the magnitude of θ, giving positive bias for θ>0 and negative bias for θ<0.^1,17^ Emerson^18^ obtained an analytical expression for $\mathrm{Bias}({\hat{\theta }}_{\text{M}LE}|\theta )$ in our setting as

(2) $$\mathrm{Bias}({\hat{\theta }}_{\mathrm{MLE}}|\theta )=\frac{I_{2}-I_{1}}{I_{2}\sqrt{I_{1}}}\{\phi (u,\theta \sqrt{I_{1}},1)-\phi (l,\theta \sqrt{I_{1}},1)\}$$

Fan et al.^15^ also provided several results on the conditional bias of the MLE.

#### 2.2.2. Mean adjusted estimators

Whitehead^19^ suggested adjusting ${\hat{\theta }}_{\text{M}LE}(k,z)$ by subtracting an estimate of its bias; this has since been referred to as a mean adjusted estimator (MAE). With the true value of θ unknown, which determines the bias of ${\hat{\theta }}_{\text{M}LE}$, a proposed estimator which we refer to as ${\hat{\theta }}_{\text{M}AE1}$ is the solution to

$${\hat{\theta }}_{\text{M}AE1}(k,z)={\hat{\theta }}_{\text{M}LE}(k,z)-\mathrm{Bias}\{{\hat{\theta }}_{\text{M}LE}|{\hat{\theta }}_{\text{M}AE1}(k,z)\}$$

That is, an estimator which has the property $E\{{\hat{\theta }}_{\text{M}LE}|{\hat{\theta }}_{\text{M}AE1}(k,z)\}={\hat{\theta }}_{\text{M}LE}(k,z)$. No closed-form solution for ${\hat{\theta }}_{\text{M}AE1}(k,z)$ exists and it must be determined using a numerical search.

A simpler alternative to ${\hat{\theta }}_{\text{M}AE1}$, which we refer to by ${\hat{\theta }}_{\text{M}AE2}$, is

$$\begin{array}{rl} {\hat{\theta }}_{\mathrm{MAE}2}(k,z) & ={\hat{\theta }}_{\text{M}LE}(k,z)-\mathrm{Bias}\{{\hat{\theta }}_{\text{M}LE}|{\hat{\theta }}_{\text{M}LE}(k,z)\}\\ & ={\hat{\theta }}_{\text{M}LE}(k,z)-\frac{I_{2}-I_{1}}{I_{2}\sqrt{I_{1}}}[\phi \{u,\theta_{\text{M}LE}(k,z)\sqrt{I_{1}},1\}\\ & \;-\phi \{l,\theta_{\text{M}LE}(k,z)\sqrt{I_{1}},1\}] \end{array}$$

The principal advantage of this estimator over ${\hat{\theta }}_{\text{M}AE1}$ is the ease of its determination, which has an explicit solution using equation (2). This estimator was considered by Guo and Liu^20^ in the context of an exact two-stage group sequential single-arm trial for a Bernoulli-distributed primary outcome.

#### 2.2.3. Median unbiased estimator

Next, we consider an MUE for θ. MUEs are dependent on the definition of an ordering of the sample space. Following Jennison and Turnbull,^1^ we write $\left(k^{\prime },z^{\prime })≻(k,z\right)$ to denote that $\left(k^{\prime },z^{\prime }\right)$ is above (k,z) in a chosen ordering of the sample space, and denote by P{(K,Z)≻(k,z)|θ} the probability of observing a result above (k,z) conditional on θ. With this, a MUE ${\hat{\theta }}_{\text{M}UE}(k,z)$ can be defined as the solution to

$$P\{(K,Z)≻(k,z)|{\hat{\theta }}_{\text{MUE}}(k,z)\}=0.5$$

Unfortunately, there is no single most-logical way to order possible outcomes of a group sequential trial.^1^ A choice must be made in practice between a number of orderings, all of which have their own appeal. Armitage^21^ and Emerson and Fleming^3^ have considered an MLE ordering. Chang^16^ considered a likelihood-ratio-based ordering. Rosner and Tsiatis^22^ considered a score test-based ordering. Respectively, these consider $\left(k^{\prime },z^{\prime })≻(k,z\right)$ if $\left(k^{\prime },z^{\prime }\right)$ results in a larger value of the MLE, likelihood-ratio, or score. For more details on these, see Jennison and Turnbull.^1^

Here, we focus on the stage-wise ordering, first proposed by Armitage,^23^ which has since been used by many authors (see, e.g., Siegmund^24^, Fairbanks and Madsen^25^, Tsiatis et al.^26^). As noted earlier, this ordering requires the continuation region to be an interval, which was our reason for the restriction to designs such that stage 2 occurs when $z_{1}\in (l,u)$. In a two-stage trial, using the stage-wise ordering, $\left(k^{\prime },z^{\prime })≻(k,z\right)$ if any of the following occurs

-
$k^{\prime }=k$ and $z^{\prime }\geq z$
-
$1=k^{\prime }<k=2$ and $z^{\prime }\geq u$, and
-
$2=k^{\prime }>k=1$ and z<l

Using this, along with the distribution of the test statistics, we have

$$P\{(K,Z)≻(k,z)|\theta \}=\left\{ \begin{array}{cc} 1-\Phi (z,\theta \sqrt{I_{1}},1) & \;:\;k=1\\ 1-\Phi (u,\theta \sqrt{I_{1}},1)+\int_{z}^{\infty }f(2,x|\theta )\;dx & \;:\;k=2 \end{array}\right.$$

From observing the form when k=1, it is clear that ${\hat{\theta }}_{\text{M}UE}(1,z)={\hat{\theta }}_{\text{M}LE}(1,z)$. However, ${\hat{\theta }}_{\text{M}UE}(2,z)$ must be determined via a numerical search.

#### 2.2.4. Uniform minimum variance unbiased estimator

A uniform minimum variance unbiased estimator (UMVUE) is an estimator of interest in most estimation problems, and the considered two-stage group sequential design framework is no exception. Emerson and Fleming^3^ proposed an unbiased estimator of θ calculated by applying the Rao-Blackwell technique to the unbiased estimate of θ formed from the stage 1 data, $Z_{1}/\sqrt{I_{1}}$. They considered (K,Z) to be a complete sufficient statistic for θ, which would mean their proposed estimator would be the UMVUE. Unfortunately, Liu and Hall^27^ later proved that (K,Z) is sufficient but not complete. They did, however, demonstrate that this estimator is uniformly minimum variance unbiased amongst a class of estimators termed truncation adaptable. Put simply, this means the class of estimators that do not require knowledge of the number of further analyses nor their associated information levels that would have occurred had the study continued past the terminal analysis K. We term this ${\hat{\theta }}_{\text{U}MVUE}$, with it’s formal definition being

$${\hat{\theta }}_{\text{U}MVUE}(k,z)=E\left(\frac{Z_{1}}{\sqrt{I_{1}}}|k,z\right)$$

In the general case of a multi-stage group sequential trial, the computation of ${\hat{\theta }}_{\text{U}MVUE}$ can be computationally intensive; Emerson^28^ and Emerson and Kittelson^29^ discuss the necessary calculations at length. In our two-stage setting, we demonstrate in the Supplemental Materials that the UMVUE can be computed as

$${\hat{\theta }}_{\text{U}MVUE}(k,z)=\left\{ \begin{array}{cc} {\hat{\theta }}_{\text{M}LE}(k,z) & \;:\;k=1\\ {\hat{\theta }}_{\text{M}LE}(k,z)-\frac{I_{2}-I_{1}}{I_{2}\sqrt{I_{1}}}\frac{\phi \{u,z\sqrt{I_{1}/I_{2}},(I_{2}-I_{1})/I_{2}\}-\phi \{l,z\sqrt{I_{1}/I_{2}},(I_{2}-I_{1})/I_{2}\}}{\Phi \{u,z\sqrt{I_{1}/I_{2}},(I_{2}-I_{1})/I_{2}\}-\Phi \{l,z\sqrt{I_{1}/I_{2}},(I_{2}-I_{1})/I_{2}\}} & \;:\;k=2 \end{array}\right.$$

By definition, $\mathrm{Bias}({\hat{\theta }}_{\text{U}MVUE}|\theta )=0$ for all θ. Note that ${\hat{\theta }}_{\text{U}MVUE}(2,z)$ takes a functional form equivalent to an adjusted version of ${\hat{\theta }}_{\text{M}LE}(2,z)$.

#### 2.2.5. Conditional maximum likelihood estimator

Highlighting the potential importance of reducing conditional bias, Liu et al.^13^ and Fan et al.^15^ considered an estimator, which we refer to as ${\hat{\theta }}_{\text{C}MLE}$, based on the conditional likelihood. Specifically, they examined

$${\hat{\theta }}_{\text{C}MLE}(k,z)=\underset{\theta }{\mathrm{argmax}}\;f_{cond}(k,z|\theta )$$

Like several of the estimators above, no closed form solution exists for ${\hat{\theta }}_{\text{C}MLE}$, though it can be readily determined using, e.g., Newton–Raphson iteration. For this approach, Liu et al.^13^ provide a number of useful results including forms for the differential of the conditional log-likelihood and the conditional Fisher’s information. As highlighted by Liu et al.,^13^ ${\hat{\theta }}_{\text{C}MLE}$ is identical to the Kth order bias adjusted estimator of Troendle and Yu.^14^ Similarly, Fan et al.^15^ demonstrated that ${\hat{\theta }}_{\text{C}MLE}$ is identical to an estimator defined as the solution to either of the following problems

$$\begin{array}{rl} E\{(K,Z)|k\} & =(k,z)\\ \hat{\theta }(k,z) & ={\hat{\theta }}_{\text{M}LE}(k,z)-\mathrm{Bias}\{{\hat{\theta }}_{\text{M}LE}|\hat{\theta }(k,z),k\} \end{array}$$

i.e., as the solution to the conditional version of the problem that defines ${\hat{\theta }}_{\text{M}AE1}$.

#### 2.2.6. Conditional weighted MAE

Shimura et al.^30^ proposed a shrinkage estimator for use when a group sequential trial terminates early. Their estimator requires a prior guess at θ; denoting this prior guess by $\theta_{0}$, Shimura et al.^30^ suggested $\theta_{0}$ be specified as the difference under which the trial was powered. Then, their estimator, which we denote by ${\hat{\theta }}_{\text{C}WMAE}$, sets

$${\hat{\theta }}_{\text{C}WMAE}(1,z)={\hat{\theta }}_{\text{M}LE}(1,z)-Bias\{{\hat{\theta }}_{\text{M}LE}|{\hat{\theta }}_{*}(1,z),1\}$$

where

$${\hat{\theta }}_{*}(1,z)=\frac{\{{\hat{\theta }}_{\text{M}LE}(1,z)-\theta_{0}{\}}^{2}}{\{{\hat{\theta }}_{\text{M}LE}(1,z)-\theta_{0}{\}}^{2}+I_{1}^{-1}}{\hat{\theta }}_{\text{M}LE}(1,z)+\left[1-\frac{\{{\hat{\theta }}_{\text{M}LE}(1,z)-\theta_{0}{\}}^{2}}{\{{\hat{\theta }}_{\text{M}LE}(1,z)-\theta_{0}{\}}^{2}+I_{1}^{-1}}\right]\theta_{0}$$

The weighting factor in ${\hat{\theta }}_{*}(1,z)$ was chosen to minimise the RMSE of ${\hat{\theta }}_{\text{C}WMAE}(1,z)$.

Shimura et al.^30^ proposed this estimator as a method of reducing conditional bias on early termination. They did not discuss a functional form for an estimate when a trial continues to stage 2. Here, to be able to compute the marginal performance of ${\hat{\theta }}_{\text{C}WMAE}$, we assume that ${\hat{\theta }}_{\text{C}WMAE}(2,z)={\hat{\theta }}_{\text{M}LE}(2,z)$. However, we note that alternative reasonable assumptions are possible and that ${\hat{\theta }}_{\text{C}WMAE}$ should be principally evaluated in terms of $Bias({\hat{\theta }}_{\text{C}WMAE}|\theta ,1)$ and $RMSE({\hat{\theta }}_{\text{C}WMAE}|\theta ,1)$.

#### 2.2.7. Conditional median unbiased estimator

A conditional MUE (CMUE) was proposed by Zhong and Prentice^31^ and Koopmeiners et al.^4^ No explicit solution exists to its value in general, but it can be determined numerically as the solution to

$$0.5=\int_{-\infty }^{z}f_{\text{c}ond}\{k,x|{\hat{\theta }}_{\text{C}MUE}(k,z)\}dx$$

In this instance, unlike for the MUE above, no ordering of the sample space is required.

#### 2.2.8. Conditional UMVUE

The final estimator we consider is the conditional uniform minimum variance unbiased estimator (CUMVUE, sometimes called the uniform minimum variance conditionally unbiased estimator). That is, an estimator ${\hat{\theta }}_{\text{C}UMVUE}$ such that $\mathrm{Bias}({\hat{\theta }}_{\text{C}UMVUE}|\theta ,2)=0$ for all θ. Such an estimator was originally considered by Cohen and Sackrowitz^32^ and has recently been extended by several authors (see, e.g., Kimani et al.^33^ and Robertson et al.^34^). It is calculated by applying Rao-Blackwell to the unbiased estimate of θ formed from the stage 2 data. Unfortunately, similar to the CWMAE, the CUMVUE does not come with an obvious implied definition of an estimate when the trial terminates at the interim analysis, which is required to compute the marginal bias and RMSE. Here, like Porcher and Desseaux,^5^ we assume that the MLE would be used, but note that other reasonable alternatives are possible. As we will see later, this is useful for comparing the CUMVUE to the CMLE in terms of the marginal bias and RMSE. With this, we prove in the Supplemental Materials that the CUMVUE can be written as

$${\hat{\theta }}_{\text{C}UMVUE}(k,z)=\left\{ \begin{array}{cc} {\hat{\theta }}_{\text{M}LE}(k,z) & \;:\;k=1\\ \left\{z\sqrt{I_{2}}-I_{1}{\hat{\theta }}_{\text{U}MVUE}(k,z)\}/(I_{2}-I_{1}\right) & \;:\;k=2 \end{array}\right.$$

### 2.3. Code

Having described each of the nine estimators that will be compared, we next proceed to present evaluations of their performance in five indicative study examples. Code to reproduce these results exactly is available at https://github.com/mjg211/article_code. In addition, estimators can be compared within the setting of a two-stage group sequential trial with a parallel two-arm design assuming normally distributed outcome data via the GUI to the OptGS^35^ package, available at https://mjgrayling.shinyapps.io/optgs/.

Note that in the majority of our examples, if the study is powered for θ=δ>0, then we evaluate estimator performance over the range θ∈[−2δ,2δ]. Typically, δ corresponds to either a minimum clinically important difference or an *a priori* anticipated effect^36^; either way, we view θ∈[−2δ,2δ] to then represent an extremely wide range of plausible effects, enabling patterns in estimator performance to become clear. In practice, when trying to choose a preferred estimator for a trial, clinical knowledge could be used to consider performance over a narrower range. We return to this point in the Discussion.

We highlight also that whilst each of our examples is given some clinical context to make them more tangible and practically useful, ultimately the performance of the estimators is only dependent on the clinical context through the specified values of θ, δ, l, u, $I_{1}$, and $I_{2}$.

### 3. Results

#### 3.1. Log-rank test for survival data in a two-arm parallel-group trial

To begin, we consider an important context: A two-arm parallel-group individually randomised trial for time-to-event data under the proportional hazards assumption. This may correspond, for example, to an oncology trial in which the objective is to ascertain improved overall survival for some new treatment (indexed E, for experimental, in what follows) compared to the current standard of care (indexed C, for control, in what follows). We, therefore, phrase the language below as if it is such an oncology trial.

Firstly, it is assumed that the hazard rate at time t for participants in arm C is h(t), and that it is λh(t) for participants in arm E. The log-rank test is a common approach to testing the null hypothesis that the hazard ratio is equal to one (i.e., λ=1). To explain this approach, following Jennison and Turnbull^1^, let $d_{k}$ be the total number of uncensored deaths observed when analysis k=1,2 is conducted. Assuming no ties, denote the survival times of these participants by $\tau_{1k}<\tau_{2k}<\cdots <\tau_{d_{k}k}$, with the $\tau_{ik}$ denoting the elapsed time between entry to the study and death. Let also the number of participants at analysis k known to have survived up to time $\tau_{ik}$ after treatment be $r_{iCk}$ and $r_{iEk}$ for arms C and E, respectively. The log-rank score statistic at the stage k analysis is then

$$S_{k}=\sum_{i=1}^{d_{k}}\left(\delta_{iEk}-\frac{r_{iEk}}{r_{iCk}+r_{iEk}}\right)$$

where $\delta_{iEk}=1$ if the death at time time $\tau_{ik}$ was on treatment E, and $\delta_{iEk}=0$ otherwise. It can be shown that

$$\hat{Var}(S_{k})=I_{k}=\sum_{i=1}^{d_{k}}\frac{r_{iCk}r_{iEk}}{(r_{iCk}+r_{iEk}{)}^{2}}$$

Next, set θ=−log(λ), such that larger values of θ correspond to improved overall survival in arm E compared to arm C. Then, for θ close to zero and sufficiently large $I_{k}$, the approximation $S_{k}∼N(-\theta I_{k},I_{k})$ can be used. This means that if $Z_{k}=-S_{k}/\sqrt{I_{k}}$, $\left\{Z_{1},Z_{2}\right\}$ has the canonical joint distribution with information levels $\left\{I_{1},I_{2}\right\}$ for θ.

To design such a trial, given the exact information levels $I_{1}$ and $I_{2}$ are not known pre-study, it is common to use the approximation $I_{k}\approx d_{k}/4$. We adopt this approach, and further suppose that the goal is to test the one-sided alternative $H_{A}\;:\;\theta >0$. We assume the desire is for a type-I error-rate of α=0.025 and a type-II error-rate of β=0.1 when θ=δ=−log(0.8)≈0.182 (i.e., to power for a hazard ratio of 0.8). We further suppose that the interim analysis will be conducted half-way through the maximal trial length in terms of the number of events observed, such that $I_{2}=2I_{1}$. We then focus on how the performance of the estimators changes when stopping for futility and efficacy is incorporated into the design ( l,u∈R,u>l), as opposed to stopping for futility only ( l∈R,u=∞). In both cases, we assume that l=−0.674. We choose this value because Φ(l,0,1)≈0.25 (i.e., to give a 25% chance of stopping for futility after stage 1 under $H_{0}$, which will in turn result in a low chance of incorrectly stopping for futility when θ=δ). In the case with efficacy stopping, u=2.157 is chosen, as this corresponds to the α-spend when using Lan and DeMets error spending approach with their Pocock-like spending function.^37^ The error spending approach can then be further used to show that to attain the overall desired error rates, the design with efficacy and futility stopping must have $I_{1}=117.5$ (i.e., the stage 1 analysis is performed after $d_{1}=470$ events). In contrast, the design with futility stopping only requires $I_{1}=105.75$ (i.e., $d_{1}=423$).

**Figure 1.**
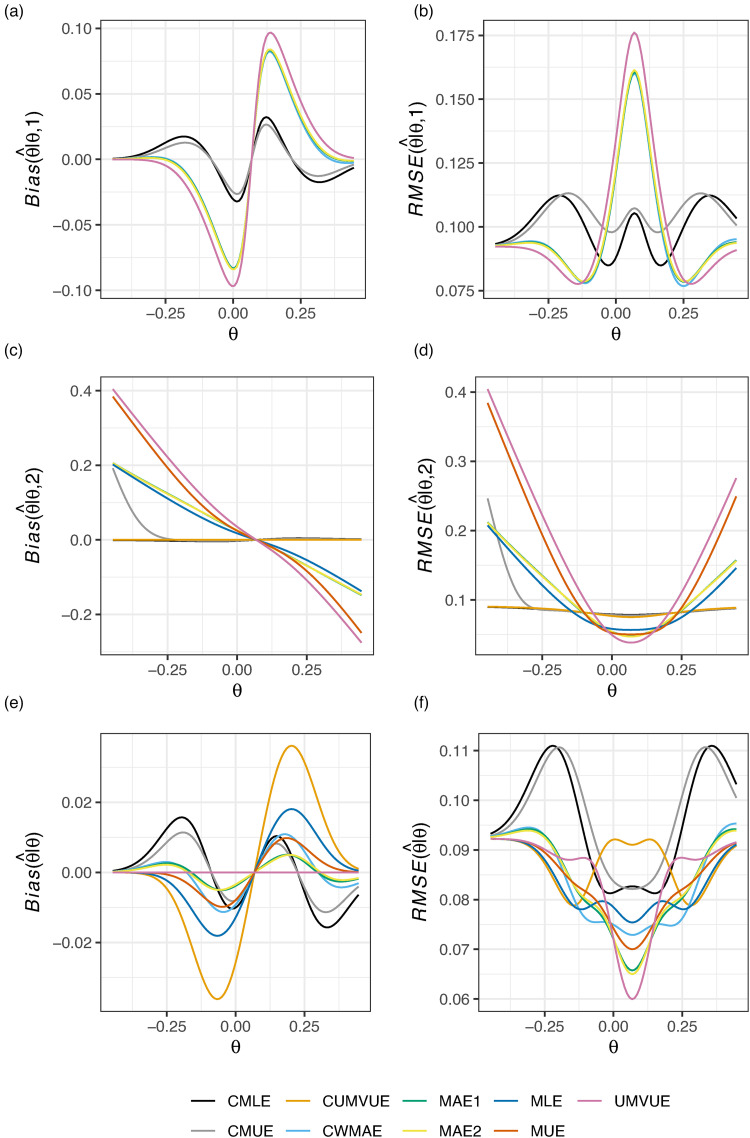
The conditional and marginal biases and residual mean square error (RMSE) of the nine considered estimators is given for Example 1: two-arm survival data, with efficacy and futility stopping.

**Figure 2.**
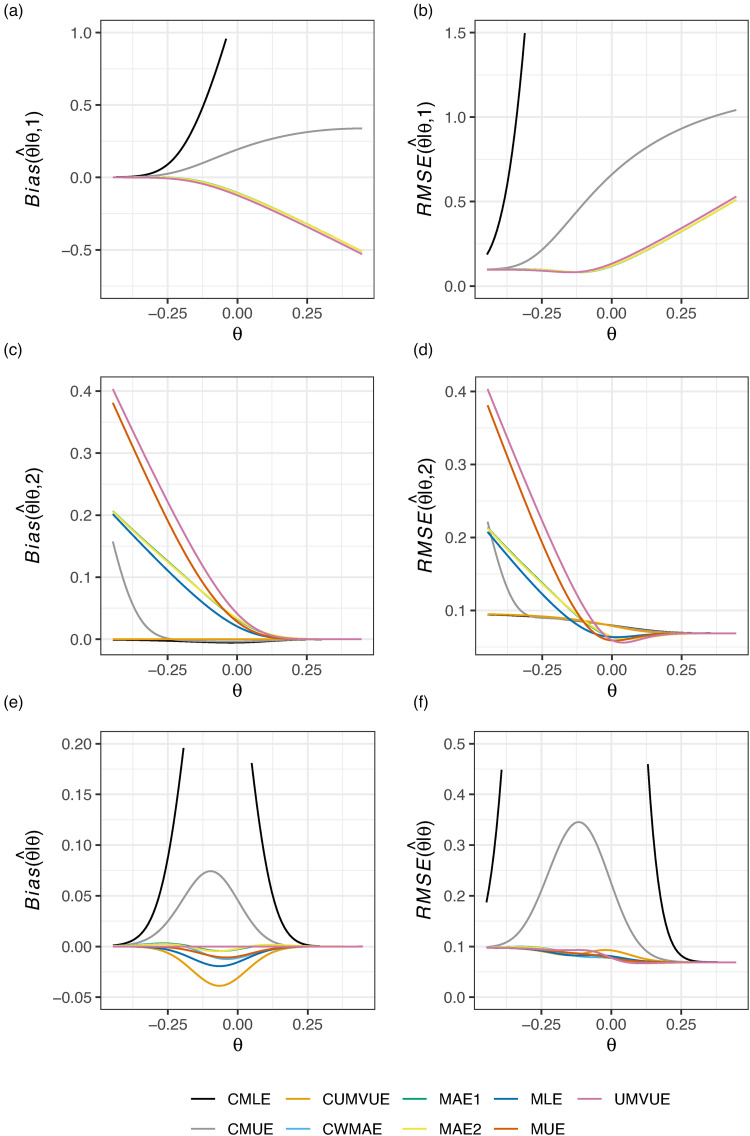
The conditional and marginal biases and residual mean square error (RMSE) of the nine considered estimators is given for Example 1: two-arm survival data, with futility stopping only. The vertical axis limits have been constrained such that CMLE performance is not visible for all θ; see Supplemental Figure S1 for a complete view.

Figure 1 then shows the performance of the estimators for the design with efficacy and futility stopping, while Figure 2 gives the corresponding results for the design with futility stopping only. In Figure 1, the CMLE and CMUE arguably have the best performance conditional on termination after stage 1. They also perform well conditional on stage 2 termination, where the CUMVUE also provides effective performance. Though the marginal RMSE of the CMLE and CMUE is sometimes larger than the other estimators (e.g., for θ∉[−δ,δ]), we may believe that they represent the most effective options in this case (particularly since it may routinely be believed that a large negative treatment effect is unlikely, so performance for negative θ may be of little concern).

However, in Figure 2, it can be seen that the marginal performance and performance conditional on termination after stage 1 of the CMLE and CMUE is extremely poor. We comment further on the CMLE’s performance in this setting in the subsequent example and in the Discussion. In the case of futility stopping only, the CUMVUE seems to be a substantially better option if one is willing to forgo a slightly larger absolute marginal bias. Alternatively, the MAEs, MAE1 and MAE2, may be preferred if the marginal bias of the CUMVUE is viewed to be too great. Contrasting the findings of Figures 1 and 2, it is thus clear that the performance of the estimators can be substantially impacted by the inclusion (or not) of efficacy stopping.

### 3.2 Single-arm phase II cancer trial with a normally distributed outcome

Wason et al.^38^ discussed the use of continuous tumour shrinkage endpoints in two-stage phase II single-arm oncology trials, for the purposes of reducing requisite sample sizes. It was assumed that outcome $X_{i}$ from participant i is distributed as $X_{i}∼N(\mu ,\sigma^{2})$, for known $\sigma^{2}$. Here, $X_{i}$ represents the percentage decrease in the sum of lesion diameters; positive values represent shrinkages in tumour size. The problem of testing $H_{0}\;:\;\theta =\mu =0$ against a one-sided alternative $H_{A}\;:\;\theta >0$ was then considered. Assuming data from $n_{k}$ patients is available at analysis k, setting

$$\begin{array}{rl} Z_{k} & =\frac{1}{\sqrt{n_{k}}\sigma^{2}}\sum_{i=1}^{n_{k}}X_{i}\\ I_{k} & =n_{k}/\sigma^{2} \end{array}$$

k=1,2, means $\left\{Z_{1},Z_{2}\right\}$ has the canonical joint distribution with information levels $\left\{I_{1},I_{2}\right\}$ for θ. The restriction was made that early stopping is only permitted for futility (i.e., u=∞), to correspond to the typical approach in phase II single-arm oncology trials. Then, assuming a desired type-I error-rate of α=0.05 and type-II error-rate of β=0.1 when θ=δ=10 (i.e., the target is 10% mean tumour shrinkage), Wason et al.^38^ found the minimax design (i.e., the design that minimises $n_{2}$) to be $n_{1}=92$, $n_{2}=139$, and l=0, on the assumption that $\sigma^{2}=1600$.

**Figure 3.**
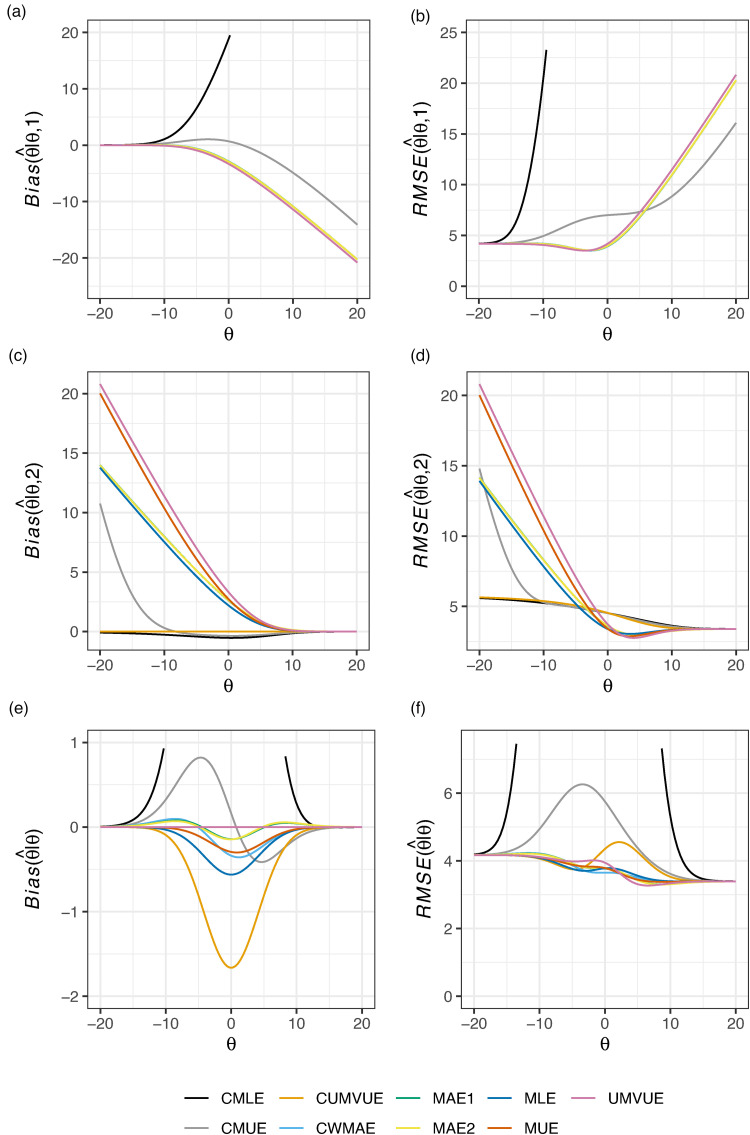
The conditional and marginal biases and residual mean square error (RMSE) of the nine considered estimators is given for Example 2: single-arm normally distributed data. The vertical axis limits have been constrained such that CMLE performance is not visible for all θ; see Supplemental Figure S2 for a complete view.

Figure 3 displays the conditional and marginal biases and RMSE of the nine considered estimators in this design when θ∈[−2δ,2δ]=[−20,20]. In this case, as in Figure 2, the CMLE is observed to have extremely large bias and RMSE, both marginally and conditionally, for certain values of θ. The UMVUE has the worst bias and RMSE is conditional on stage 2 termination. The CMUE has strong conditional performance, but fares badly marginally. MAE1 and MAE2 reduce the marginal bias compared to the MLE, without compromising on conditional performance or marginal RMSE. Thus, for this example, we may prefer either MAE1 or MAE2 if we are unsatisfied with the UMVUE’s performance conditional on stage 2 termination.

### 3.3. Crossover trial

Jones and Kenward^39^ considered sample size calculation for a 2×2×2 placebo-controlled double-blind crossover trial to evaluate the efficacy of an inhaled drug given twice daily via an inhaler in patients with chronic obstructive pulmonary disease (see Sections 2.1 and 2.4 of Jones and Kenward^39^). The primary outcome was the mean morning expiratory flow rate; outcome $X_{ijl}$ from patient $l=1,\ldots ,n_{i}$, in period j=1,2, on sequence i=1,2, was assumed to be distributed as $X_{ijl}∼N(\mu +\pi_{j}+\tau_{\text{d}(i,j)}+s_{il},\sigma_{e}^{2})$. Here, $\sigma_{e}^{2}$ reflects the known within-patient variance, μ is an intercept term, $\pi_{j}$ is an effect associated with period j, $\tau_{\text{d}(i,j)}$ is a direct treatment effect associated with the treatment applied in period j of sequence i, and $s_{il}$ is an effect associated with the lth subject on sequence i. Labelling the treatments such that d(i,j)=A,B, assuming that sequences 1 and 2 are AB and BA respectively, the problem of testing $H_{0}\;:\;\theta =\tau_{B}-\tau_{A}=0$ was considered. Suppose that at analysis k, $n_{1}=n_{2}=kn$. Then, setting

$$\begin{array}{rl} Z_{k} & =\frac{1}{2}\sqrt{\frac{kn}{\sigma_{e}^{2}}}\sum_{l=1}^{kn}(X_{1l}-X_{2l})\\ I_{k} & =kn/\sigma_{e}^{2}\\ X_{il} & =X_{i2l}-X_{i1l} \end{array}$$

it can be shown that $\left\{Z_{1},Z_{2}\right\}$ has the canonical joint distribution with information levels $\left\{I_{1},I_{2}\right\}$ for θ^1,39,40^. Here, we again assume a one-sided alternative $H_{A}\;:\;\theta >0$. Following Jones and Kenward^39^, we assume that the desired type-I error-rate is α=0.05 and type-II error-rate is β=0.2 when θ=δ=10, further supposing that $\sigma_{e}^{2}=326$. Then, if power-family stopping boundaries^41^ are used with shape parameter −0.25, the resulting two-stage design has n=10.384, l=0.269, and u=2.730.

**Figure 4.**
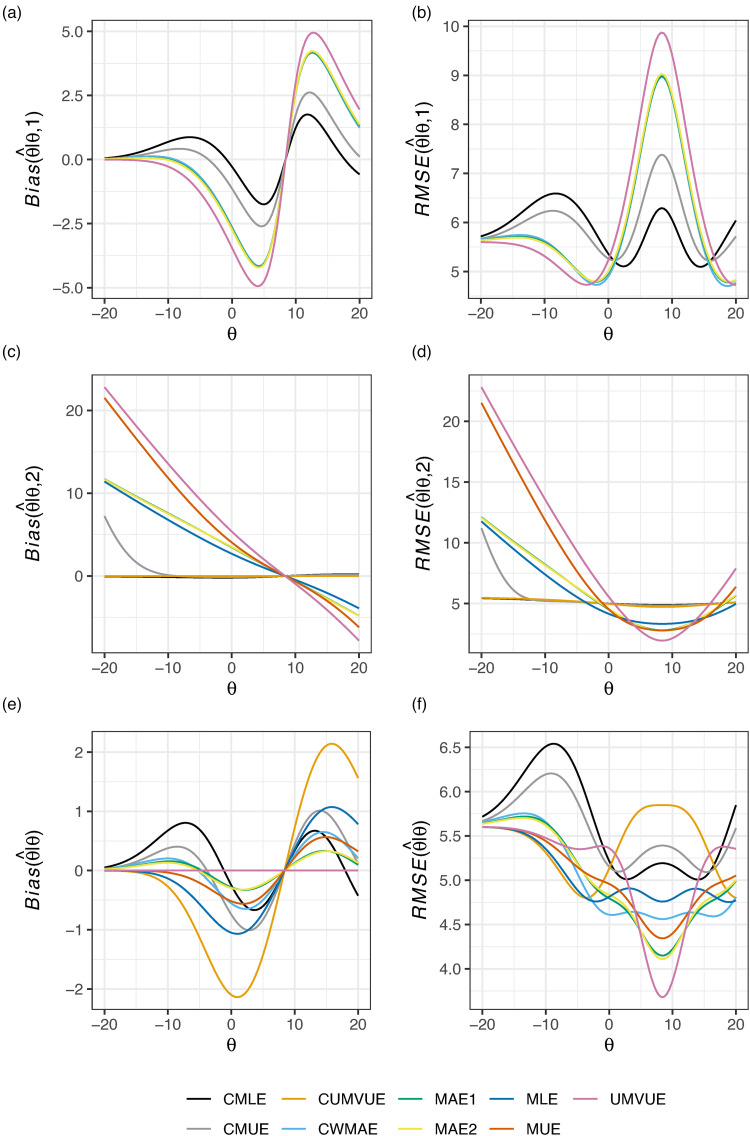
The conditional and marginal biases and residual mean square error (RMSE) of the nine considered estimators is given for Example 3: crossover data.

Figure 4 displays the conditional and marginal biases and RMSE of the nine considered estimators in this design when θ∈[−2δ,2δ]=[−20,20]. The results are overall similar to those given in Figure 1. The CMLE and CMUE perform well conditionally, but arguably not marginally, particularly in terms of their marginal RMSE. Here, it is clearer that the MUE seemingly trades an improvement in the conditional bias, conditional RMSE, and marginal RMSE, for a larger marginal bias. The CWMAE has particularly effective performance in terms of its marginal RMSE; whilst other estimators have lower marginal RMSE in the region around θ=9, it maintains strong performance over a wider range of θ. Given it also performs well in terms of its marginal bias and conditional bias/RMSE, it may be the best candidate for use in this case.

### 3.4. Single-arm phase II cancer trial with a Bernoulli distributed outcome

Above, we have covered examples with time-to-event and normally distributed data. Here, we focus on a case with Bernoulli data. Schoffski et al.^42^ presented the results of a phase II single-arm oncology trial conducted to assess the activity of crizotinib in patients with advanced clear-cell sarcoma with MET alterations. Tumour response was used as the primary outcome and thus it was assumed that outcome $X_{i}$ from participant i was distributed as $X_{i}∼Bern(\pi )$, for response rate π∈[0,1]. Simon’s two-stage design was used, with the design specifying termination for futility after 12 patients if 1 or fewer participant exhibited a response (i.e., if $\sum_{i=1}^{12}X_{i}\leq 1$). Otherwise, a further 23 participants were to be recruited. These parameters were chosen to detect an improvement from a 10% response rate to a 30% response rate, with type-I and type-II error-rates of 10%.

This design can be mapped to our setting as follows (see section 3.6 of Jennison and Turnbull^1^ for further details). First, set $H_{0}\;:\;\theta =\pi -\pi_{0}=0$, where $\pi_{0}\in (0,1)$ is a specified clinically uninteresting response rate. Denote by $n_{k}$ the number of participant responses available at analysis k and set

$$\begin{array}{rl} Z_{k} & =\left(\frac{1}{n_{k}}\sum_{i=1}^{n_{k}}X_{i}-\pi_{0}\right)\sqrt{I_{k}}\\ I_{k} & =\frac{n_{k}}{\pi_{0}(1-\pi_{0})} \end{array}$$

Then, for π close to $\pi_{0}$, $\left\{Z_{1},Z_{2}\right\}$ approximately has the canonical joint distribution with information levels $\left\{I_{1},I_{2}\right\}$ for θ.

The design from Schoffski et al.^42^ corresponds to u=∞, with $\pi_{0}=0.1$, $n_{1}=12$, $n_{2}=35$, $H_{A}\;:\;\theta >0$, and

$$\begin{array}{rl} l & =\left(\frac{1}{12}-0.1\right)\sqrt{I_{1}}\approx -0.192\\ I_{1} & =\frac{12}{0.1\times 0.9}\approx 133.3\\ I_{2} & =\frac{35}{0.1\times 0.9}\approx 388.9 \end{array}$$

Finally, the study was powered for θ=δ=0.3−0.1=0.2.

**Figure 5.**
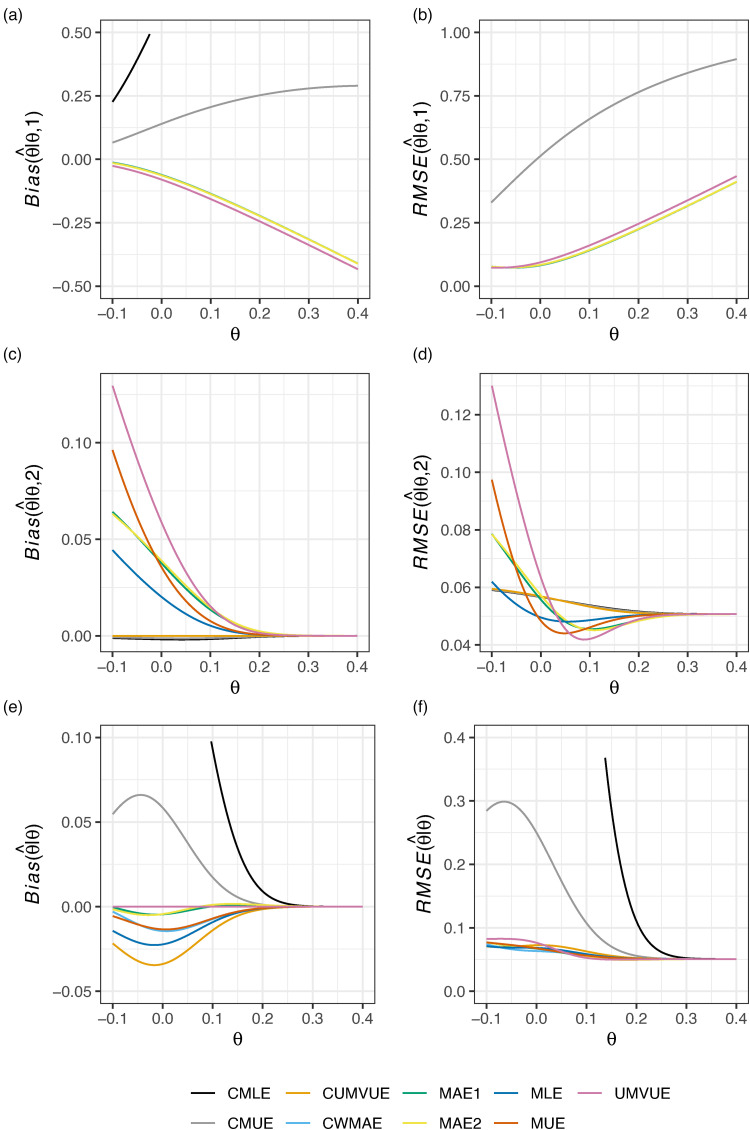
The conditional and marginal biases and residual mean square error (RMSE) of the nine considered estimators is given for Example 4: single-arm Bernoulli distributed data. The vertical axis limits have been constrained such that CMLE performance is not visible for all θ; see Supplemental Figure S3 for a complete view.

Figure 5 shows the nine estimators’ performance in this setting, for θ∈[−0.1,0.4], to correspond to π∈[0,0.5], where the upper limit 0.5 is chosen as a sufficiently large response rate that is unlikely to be observed in practice given the study was powered at π=0.3. Once more, we see that u=∞ results in extremely poor performance of the CMLE, and to a slightly lesser extent also the CMUE. Here, there is arguably only a direct choice to be made between either MAE1/MAE2 (if one is more concerned about marginal performance) or the MLE/CUMVUE (if one is more concerned about performance conditional on stage 2 termination). The UMVUE is, as always, optimal if one cares only about marginal bias. However, it fares particularly poorly conditionally in this case.

Note that we comment in the Discussion on the use of the canonical joint distribution framework for Bernoulli data.

### 3.5. Matched pairs trial

We conclude with an example with a two-sided alternative hypothesis. Jennison and Turnbull^1^ discussed (see Sections 3.1.3 and 3.2.2 of their book) the sequential design of a matched pairs trial in which subjects are paired so that those in the same pair have similar values of important prognostic factors. Matched pairs designs are also commonly employed in paired-eye and paired-teeth trials, as well as twin studies. One subject in each pair is randomly allocated treatment A and the other receives treatment B; letting $X_{Ai}$ and $X_{Bi}$ denote the responses of the subject in pair i who receive treatments A and B respectively, it was assumed that $X_{Ai}-X_{Bi}∼N(\mu_{A}-\mu_{B},{\tilde{\sigma }}^{2})$ with ${\tilde{\sigma }}^{2}$ known. The problem of testing $H_{0}\;:\;\theta =\mu_{A}-\mu_{B}=0$ against $H_{A}\;:\;\theta \neq 0$ was considered. If $n_{k}$ pairs of observations are available at analysis k, it was shown that

$$\begin{array}{rl} Z_{k} & =\frac{1}{\sqrt{n_{k}}{\tilde{\sigma }}^{2}}\sum_{i=1}^{n_{k}}(X_{Ai}-X_{Bi})\\ I_{k} & =n_{k}/{\tilde{\sigma }}^{2} \end{array}$$

k=1,2, results in $\left\{Z_{1},Z_{2}\right\}$ having the canonical joint distribution with information levels $\left\{I_{1},I_{2}\right\}$ for θ. Assuming a desired type-I error-rate of α=0.05 and type-II error-rate of β=0.1 when θ=±δ=±1, the following information levels are required when utilising O’Brien-Fleming^43^ stopping boundaries at equally spaced information levels $I_{k}=5.292k$. Thus, on the supposition that ${\tilde{\sigma }}^{2}=6$, $n_{k}=31.751k$, i.e., 31.751 pairs of observations are required per stage of the trial. Furthermore, the trial continues to stage 2 when $z_{1}\in (-2.796,2.796)$.

**Figure 6.**
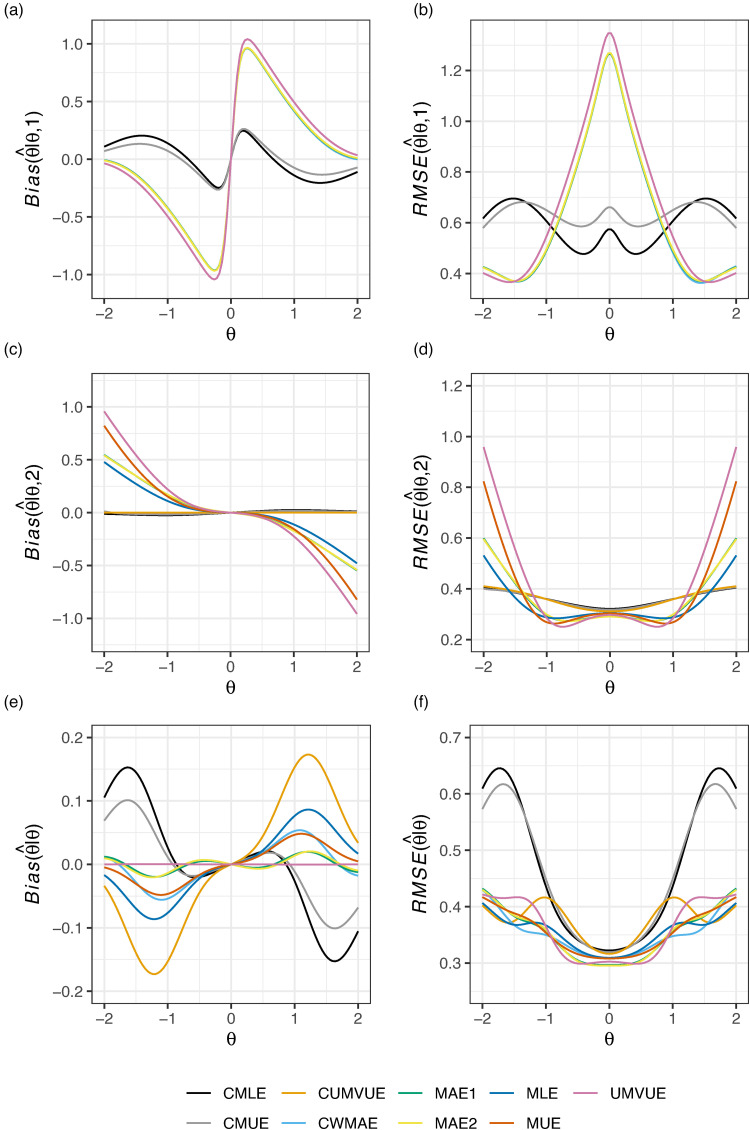
The conditional and marginal biases and residual mean square error (RMSE) of the nine considered estimators is given for Example 5: matched pairs data.

Figure 6 displays the conditional and marginal biases and RMSE of the nine considered estimators in this design when θ∈[−2δ,2δ]=[−2,2]. It displays rotational symmetry about θ=0 owing to the use of O’Brien-Fleming^43^ boundaries. Observe that the UMVUE performs worst in terms of conditional bias and RMSE. The CMLE and CMUE arguably perform best in terms of $\mathrm{Bias}(\hat{\theta }|\theta ,1)$ and $\mathrm{RMSE}(\hat{\theta }|\theta ,1)$. Though, like in Figure 1, their additional strong performance conditional on stage 2 termination does not translate to effective marginal performance. The MAE estimators, as in all previous examples, have almost identical performance. The MAE and CWMAE estimators perform similarly conditional on interim termination, but the CWMAE performs slightly better conditional on progression to stage two, and slightly worse marginally. The CMLE and CUMVUE have almost identical values for $\mathrm{Bias}(\hat{\theta }|\theta ,2)$ and $\mathrm{RMSE}(\hat{\theta }|\theta ,2)$, but their different approaches after stage 1 termination lead to substantially different marginal bias and RMSE. In this example, it is likely either the CUMVUE should be preferred if one is primarily concerned about performance conditional on stage 2 termination, or one of the MAE estimators should be used if both marginal and conditional bias/RMSE are of sizeable concern.

## 4. Discussion

In this paper, we have compared the performance of nine estimators for the principal parameter of interest after a two-stage group sequential trial, within the context of five example trials, evaluating their conditional and marginal biases and RMSE. Unfortunately, as is clear from Figures 1 to 6, there is no single estimator that performs best for the conditional and marginal biases and RMSE. However, a number of recommendations remain possible. Firstly, if one cares solely about the marginal bias, the UMVUE is naturally the optimal estimator. Secondly, if only bias on termination after the interim analysis is of concern, as it may be in the case where interim stopping is only allowed for futility, then the CUMVUE should likely be preferred. Both of these estimators are observed to perform poorly by some measures, though. In particular, as has been discussed previously, the UMVUE often has large conditional bias and RMSE.^13–15^ In general, we would also caution against focusing solely on a single measure of estimator performance. For example, even on termination for futility after stage 1, effective estimation may still be important for decision-making about subsequent studies. Thirdly, the use of the CMLE (and to a lesser extent the CMUE) is not advisable in certain settings due to its large bias. This result about the CMLE was recently formally proved by Berckmoes et al.,^44^ who demonstrated for the early stopping boundaries used in the second example ( l=0, u=∞) that the bias of the CMLE is unbounded. We elaborate on this further in the Supplemental Materials by giving an example of the conditional log-likelihood.

Where the conditional and marginal values of the bias and RMSE are all of importance, arguably the MAEs may be considered the best choice as they are typically amongst the better estimators on all six sub-panels of Figures 1 to 6. However, an additional potentially important consideration when choosing an estimator, beyond their bias and RMSE, is the predictability of the information levels. The MAE estimates of θ after stage 1 are dependent on the value of $I_{2}$, which may be undesirable if $I_{2}$ is unpredictable.^1^ It was for this reason the stage-wise ordering was used to compute a MUE; this ensures that ${\hat{\theta }}_{\text{M}UE}(1,z)$ is independent of $I_{2}$. Such unpredictability in information levels may arise, for example, when the information levels are dependent on the unknown parameter of interest (e.g., for Bernoulli data in a randomised trial). In this setting, there is good reason to not utilise the MAEs. In contrast, in our opinion the computational complexity of the considered estimators is not a good justification for choosing a particular estimation procedure. Explicit formulae are given here for a number of estimators, and even those requiring numerical methods of determination are dependent only on a one-dimensional search which can be readily performed.

Overall, given the performance of the estimators is highly dependent on the underlying design, we would thus always recommend evaluations be performed to help choose the estimators for any proposed group sequential design. When this is not possible, our simple recommendation would be to utilise one of the MAE estimators in the case that information levels are predictable. When the information levels are not predictable, the CWMAE may be a fallback choice, owing to its effective performance across the sub-panels in the figures given here. Such recommendations, however, are based on surveying performance across a wide range of values of θ. In practice, it would be logical to use available clinical knowledge to help better target the range of θ used to determine the preferred estimator. We focused on performance for θ∈[−2δ,2δ], where δ is the target difference at which the study is powered. In some trials, it may be the case that the treatments under investigation make a negative value of θ unlikely. In addition, θ close to twice the target difference would be a rare find. The region θ∈[0,δ] may be an effective narrower range to consider in general; when this is the case, our primary recommendations around the use of the MAE estimators or CWMAE above do not change. However, it may be more common to find the conditional performance of the UMVUE palatable; in Figure 2, for example, considering only θ∈[0,δ]=[0,10], the UMVUE may be far more easily argued to be the best choice.

We acknowledge some limitations to our work. Firstly, though we have considered a large number of possible estimators, others have been proposed in the literature. In particular, Wang and Leung^45^ proposed the use of parametric-bootstrapping procedures to attain a bias-adjusted estimator. We omit consideration of it here due to the computational complexity involved in evaluating their approach across numerous values of θ in several trial examples. Additionally, Zhong and Prentice^31^ proposed an estimator that is a weighted combination of the stage-wise MLEs. We omit it here as the stage two estimate is dependent on both $z_{1}$ and z, not simply z as for all the estimators considered in our work. We also considered only two-stage group sequential designs; it cannot be guaranteed that our recommendations above would hold true for designs with a larger number of stages. Furthermore, we are unavoidably only able to compare performance of the estimators across a small number of the infinite possible trial scenarios. We note again that the best estimator for a given trial will be (as discussed) design dependent; thus all conclusions come with the caveat that they should be investigated further for any specific parameters of interest. Our available R code, and implementation in a GUI for parallel arm trials with normal outcome data, may assist heavily with this task.

A reviewer raised an interesting comment in regard to the trial scenarios considered, about how point estimation may be affected by the choice of stopping boundaries (e.g., O’Brien-Fleming, Pocock). In general, it is reasonable to anticipate that more ‘aggressive’ stopping boundaries (i.e., boundaries that increase the probability of termination after stage 1) will result in reduced values of $\mathrm{Bias}(\hat{\theta }|\theta ,1)$ and $\mathrm{RMSE}(\hat{\theta }|\theta ,1)$, but increased values of $\mathrm{Bias}(\hat{\theta }|\theta ,2)$ and $\mathrm{RMSE}(\hat{\theta }|\theta ,2)$, everything else being equal. The effect on marginal performance is more challenging to predict; for some θ it will improve, for others it will be worse. We illustrate an example of this in the Supplemental Materials, providing results for the fifth example on matched pairs data, but where the stopping boundaries are Pocock rather than O’Brien-Fleming. In general, we believe it is likely that the impact of the stopping boundaries on the study’s maximal and expected sample sizes will be the principal driver of the choice of bounds, as opposed to their effect on inference. However, when a choice must be made between candidate group sequential designs that are otherwise considered to have similar advantages/disadvantages, the consequences for inference may be a useful additional consideration. Bowden and Wason^46^ have considered this in the context of exact single-arm phase II oncology trial designs for Bernoulli data, determining optimised combinations of design and analysis procedure.

We also end with caution against the use of the canonical joint distribution and the estimators given here in some settings. In particular, though we included an example with Bernoulli data to illustrate how the methodology can be applied in that setting, for such studies designs leveraging exact binomial densities and associated estimators should likely be preferred for all but the largest trial sample sizes (see, e.g., Porcher and Desseaux^5^ for the single-arm case, while Bibbona and Rubba^47^ provide relevant results for other designs). In addition, for time-to-event data in small-sample settings, direct simulation of study data and evaluation of estimator performance in this way (rather than using the canonical approximation) would be advisable.

In conclusion, the best estimator for a given trial is dependent on the estimator’s relative performance for, and the relative desire to minimise each of, the conditional and marginal biases and RMSE. Evaluating the performance of each of the estimators in a given trial design scenario can be efficiently completed. Undertaking this task in practice will enable investigators to make a more effective choice on how to estimate their parameter of interest.^7^ To date, few group sequential trials have computed adjusted estimates,^7,8^ which may be particularly problematic for their subsequent inclusion in meta-analyses. It may also negatively impact decision-making around whether a treatment should be further developed. We encourage their increased use in future studies.

## Supplemental Material

sj-pdf-1-smm-10.1177_09622802221137745 - Supplemental material for Point estimation following a two-stage group sequential trial Click here for additional data file. Supplemental material, sj-pdf-1-smm-10.1177_09622802221137745 for Point estimation following a two-stage group sequential trial by Michael J Grayling and James MS Wason in Statistical Methods in Medical Research

## References

[1] Jennison C Turnbull B. Group sequential methods with applications to clinical trials. New York, NY: Chapman and Hall/CRC, 2000.

[2] Whitehead J. The design and analysis of sequential clinical trials. Chichester, UK: Wiley, 1997.

[3] Emerson S Fleming T. Parameter estimation following group sequential hypothesis testing. Biometrika 1990; 77: 875–892.

[4] Koopmeiners J Feng Z Pepe M. Conditional estimation after a two-stage diagnostic biomarker study that allows early termination for futility. Stat Med 2012; 31: 420–435. 22238117 10.1002/sim.4430 PMC3641588

[5] Porcher R Desseaux K. What inference for two-stage phase II trials? BMC Med Res Methodol 2012; 12: 117. 22867439 10.1186/1471-2288-12-117 PMC3445829

[6] Shimura M Goshoc M Hirakawa A. Comparison of conditional bias-adjusted estimators for interim analysis in clinical trials with survival data. Stat Med 2017; 36: 2067–2080. 28211076 10.1002/sim.7258

[7] Robertson D Choodari-Oskooei B Dimairo M, et al. Point estimation for adaptive trial designs. *arXiv* 2021; 2105.08836.

[8] Zhang J Blumenthal G He K, et al. Overestimation of the effect size in group sequential trials. Clin Cancer Res 2012; 18: 4872–4876. 22753584 10.1158/1078-0432.CCR-11-3118

[9] Stevely A Dimairo M Todd S, et al. An investigation of the shortcomings of the CONSORT 2010 statement for the reporting of group sequential randomised controlled trials: A methodological systematic review. PLoS ONE 2015; 10: 1–20. 10.1371/journal.pone.0141104 PMC4631356 26528812

[10] Blackwell D. Conditional expectation and unbiased sequential estimation. Annals Math Stat 1947; 18: 105–110.

[11] Lehmann E Stein C. Completeness in the sequential case. Ann Math Stat 1950; 21: 376–385.

[12] Armitage P McPherson C Rowe B. Repeated significance tests on accumulating data. J R Stat Soc A 1969; 132: 235–244.

[13] Liu A Troendle J Yu K, et al. Conditional maximum likelihood estimation following a group sequential test. Biom J 2004; 46: 760–768.

[14] Troendle J Yu K. Conditional estimation following a group sequential clinical trial. Commun Stat - Theory Methods 1999; 28: 1617–1634.

[15] Fan X DeMets D Lan K. Conditional bias of point estimates following a group sequential test. J Biopharm Stat 2004; 14: 505–530. 15206542 10.1081/bip-120037195

[16] Chang M. Confidence intervals for a normal mean following a group sequential test. Biometrics 1989; 45: 247–254. 2720054

[17] Pinheiro J DeMets D. Estimating and reducing bias in group sequential designs with Gaussian independent increment structure. Biometrika 1997; 84: 831–845.

[18] Emerson S. *Parameter estimation following group sequential hypothesis testing*. PhD Thesis, University of Washington, Seattle, 1988.

[19] Whitehead J. On the bias of maximum likelihood estimation following a sequential test. Biometrika 1986; 73: 573–581.

[20] Guo H Liu A. A simple and efficient bias-reduced estimator of response probability following a group sequential phase ii trial. J Biopharm Stat 2005; 15: 773–781. 16078384 10.1081/BIP-200067771

[21] Armitage P. Numerical studies in the sequential estimation of a binomial parameter. Biometrika 1958; 45: 1–15.

[22] Rosner G Tsiatis A. Exact confidence intervals following a group sequential trial: A comparison of methods. Biometrika 1988; 75: 723–729.

[23] Armitage P. Restricted sequential procedures. Biometrika 1957; 44: 9–56.

[24] Siegmund P. Estimation following sequential tests. Biometrika 1978; 65: 341–349.

[25] Fairbanks K Madsen R. P values for tests using a repeated significance test design. Biometrika 1982; 69: 69–74.

[26] Tsiatis A Rosner G Mehta C. Exact confidence intervals following a group sequential test. Biometrics 1984; 40: 797–803. 6518248

[27] Liu A Hall W. Unbiased estimation following a group sequential test. Biometrika 1999; 86: 71–78.

[28] Emerson S. Computation of the uniform minimum variance unbiased estimator of a normal mean following a group sequential trial. Comput Biomed Res 1993; 26: 68–73. 8444028 10.1006/cbmr.1993.1004

[29] Emerson S Kittelson J. A computationally simpler algorithm for the umvue of a normal mean following a group sequential trial. Biometrics 1997; 53: 365–369. 9147601

[30] Shimura M Maruo K Gosho M. Conditional estimation using prior information in 2-stage group sequential designs assuming asymptotic normality when the trial terminated early. Pharm Stat 2018; 17: 400–413. 29687592 10.1002/pst.1859

[31] Zhong H Prentice R. Bias-reduced estimators and confidence intervals for odds ratios in genome-wide association studies. Biostatistics 2008; 9: 621–634. 18310059 10.1093/biostatistics/kxn001 PMC2536726

[32] Cohen A Sackrowitz H. Two stage conditionally unbiased estimators of the selected mean. Stat Probab Lett 1989; 8: 273–278.

[33] Kimani P Todd S Stallard N. Conditionally unbiased estimation in phase II/III clinical trials with early stopping for futility. Stat Med 2013; 32: 2893–2910. 23413228 10.1002/sim.5757 PMC3813981

[34] Robertson D Prevost A Bowden J. Unbiased estimation in seamless phase II/III trials with unequal treatment effect variances and hypothesis-driven selection rules. Stat Med 2016; 35: 3907–3922. 27103068 10.1002/sim.6974 PMC5026174

[35] Wason J. OptGS: An R package for finding near-optimal group-sequential designs. J Stat Softw 2015; 66: 1–13.

[36] Kunzmann K Grayling M Lee K, et al. A review of bayesian perspectives on sample size derivation for confirmatory trials. Am Stat 2021; 75: 424–432. 34992303 10.1080/00031305.2021.1901782 PMC7612172

[37] Lan K DeMets D. Discrete sequential boundaries for clinical trials. Biometrika 1983; 70: 659–663.

[38] Wason J Mander A Eisen T. Reducing sample sizes in two-stage phase II cancer trials by using continuous tumour shrinkage end-points. Eur J Cancer 2011; 47: 983–989. 21239164 10.1016/j.ejca.2010.12.007

[39] Jones B Kenward M. Design and analysis of cross-over trials. Boca Raton, FL: CRC Press, 2014.

[40] Grayling M Wason J Mander A. Group sequential crossover trial designs with strong control of the familywise error rate. Seq Anal 2018; 37: 174–203. 30393467 10.1080/07474946.2018.1466528 PMC6199128

[41] Pampallona S Tsiatis A. Group sequential designs for one-sided and two-sided hypothesis testing with provision for early stopping in favor of the null hypothesis. J Stat Plan Inference 1994; 42: 19–35.

[42] Schoffski P Wozniak A Stacchiotti S, et al. Activity and safety of crizotinib in patients with advanced clear-cell sarcoma with MET alterations: European organization for research and treatment of cancer phase II trial 90101 ‘CREATE’. Ann Oncol 2017; 28: 3000–3008. 28950372 10.1093/annonc/mdx527 PMC5834120

[43] O’Brien P Fleming T. A multiple testing procedure for clinical trials. Biometrics 1979; 35: 549–556. 497341

[44] Berckmoes B Ivanova A Molenberghs G. Conditional bias reduction can be dangerous: A key example from sequential analysis. *arXiv* 2018; 1812.06046.

[45] Wang Y Leung D. Bias reduction via resampling for estimation following sequential tests. Seq Anal 1997; 16: 249–267.

[46] Bowden J Wason J. Identifying combined design and analysis procedures in two-stage trials with a binary end point. Stat Med 2012; 31: 3874–3884. 22786815 10.1002/sim.5468 PMC3546375

[47] Bibbona E Rubba A. Boundary crossing random walks, clinical trials, and multinomial sequential estimation. Seq Anal 2012; 31: 99–107.

