# Supplementary material for: Point estimation following a two-stage group sequential trial
Source: Stat Methods Med Res. 2022 Nov 16;32(2):287–304. doi: 10.1177/09622802221137745 (PMC9896306; doi:10.1177/09622802221137745)
Supplement: sj-pdf-1-smm-10.1177_09622802221137745 - Supplemental material for Point estimation following a two-stage group sequential trial [file sj-pdf-1-smm-10.1177_09622802221137745.pdf]

Supplementary materials to 'Point estimation following a two-stage group sequential trial'

Derivation of $f(2, z|\theta)$

Consider the following simplification of the integrand of the first form given for $f(2, z|\theta)$ in the main manuscript

$$\begin{aligned}
&\sqrt{I_2}\phi(z_1, \theta\sqrt{I_1}, 1)\phi\{z\sqrt{I_2} - z_1\sqrt{I_1}, \theta(I_2 - I_1), I_2 - I_1\},\\
&= \sqrt{I_2}\frac{1}{\sqrt{2\pi}}\exp\left\{-\frac{(z_1 - \theta\sqrt{I_1})^2}{2}\right\}\frac{1}{\sqrt{2\pi}\sqrt{I_2 - I_1}}\exp\left[-\frac{\{(z\sqrt{I_2} - z_1\sqrt{I_1}) - \theta(I_2 - I_1)\}^2}{2(I_2 - I_1)}\right],\\
&= \frac{1}{\sqrt{2\pi}}\frac{1}{\sqrt{2\pi}}\sqrt{\frac{I_2}{I_2 - I_1}}\exp\left[-\frac{(z_1 - \theta\sqrt{I_1})^2(I_2 - I_1) + \{(z\sqrt{I_2} - z_1\sqrt{I_1}) - \theta(I_2 - I_1)\}^2}{2(I_2 - I_1)}\right],\\
&= \frac{1}{\sqrt{2\pi}}\frac{1}{\sqrt{2\pi}}\sqrt{\frac{I_2}{I_2 - I_1}}\exp\left[-\frac{I_2z_1^2 - 2z\sqrt{I_1I_2}z_1 + \{\theta^2(I_2 - I_1)^2 + \theta^2I_2(I_2 - I_1) + z^2I_2 - 2z\theta\sqrt{I_2}(I_2 - I_1)\}}{2(I_2 - I_1)}\right],\\
&= \frac{1}{\sqrt{2\pi}}\frac{1}{\sqrt{2\pi}}\sqrt{\frac{I_2}{I_2 - I_1}}\exp\left[-\frac{(z_1 - z\sqrt{I_1/I_2})^2}{2(I_2 - I_1)/I_2}\right]\exp\left[-\frac{\theta^2(I_2 - I_1)^2 + \theta^2I_2(I_2 - I_1) + z^2I_2 - 2z\theta\sqrt{I_2}(I_2 - I_1) - z^2I_1}{2(I_2 - I_1)}\right],\\
&= \frac{1}{\sqrt{2\pi}}\frac{1}{\sqrt{2\pi}}\sqrt{\frac{I_2}{I_2 - I_1}}\exp\left[-\frac{(z_1 - z\sqrt{I_1/I_2})^2}{2(I_2 - I_1)/I_2}\right]\exp\left[-\frac{(z - \theta\sqrt{I_2})^2}{2}\right], \qquad (3)\\
&= \frac{1}{\sqrt{2\pi}}\exp\left[-\frac{(z - \theta\sqrt{I_2})^2}{2}\right]\phi\{z_1, z\sqrt{I_1/I_2}, (I_2 - I_1)/I_2\}. \qquad (4)
\end{aligned}$$

Thus, applying Equation (4), we have

$$\begin{aligned}
f(2, z|\theta) &= \sqrt{I_2}\int_l^u \phi(z_1, \theta\sqrt{I_1}, 1)\phi\{z\sqrt{I_2} - z_1\sqrt{I_1}, \theta(I_2 - I_1), I_2 - I_1\}\,\mathrm{d}z_1,\\
&= \frac{1}{\sqrt{2\pi}}\exp\left[-\frac{(z - \theta\sqrt{I_2})^2}{2}\right]\int_l^u \phi\{z_1, z\sqrt{I_1/I_2}, (I_2 - I_1)/I_2\}\,\mathrm{d}z_1,\\
&= \frac{e^{-(z-\theta\sqrt{I_2})^2/2}}{\sqrt{2\pi}}[\Phi\{u, z\sqrt{I_1/I_2}, (I_2 - I_1)/I_2\} - \Phi\{l, z\sqrt{I_1/I_2}, (I_2 - I_1)/I_2\}], \qquad (5)
\end{aligned}$$

as stated in the main manuscript.

Derivation of $\hat{\theta}_{\text{UMVUE}}$

It is well known that $\hat{\theta}_{\text{UMVUE}}(1, z) = z/\sqrt{I_1}$, thus we need only prove the stated form for $\hat{\theta}_{\text{UMVUE}}(2, z)$. Given the formal definition of $\hat{\theta}_{\text{UMVUE}}$, to do this we require the density of z_1 conditional on $(K, Z) = (2, z)$. Observe that this is

$$f(z_1|2, z, \theta) = \frac{f(1, z_1|\theta)f(2, z|z_1, \theta)}{f(2, z|\theta)},$$

where $f(2, z|z_1, \theta)$ is the conditional density under θ of outcomes terminating at stage 2, given that $Z_1 = z_1$. Using this, we have

$$\begin{aligned}
\hat{\theta}_{\text{UMVUE}}(2, z) &= \frac{\int_l^u \frac{z_1}{\sqrt{I_1}} f(1, z_1|\theta) f(2, z|z_1, \theta) \, \mathrm{d}z_1}{f(2, z|\theta)}, \\
&= \frac{\int_l^u \frac{z_1}{\sqrt{I_1}} \sqrt{I_2}\phi(z_1, \theta\sqrt{I_1}, 1)\phi\{z\sqrt{I_2} - z_1\sqrt{I_1}, \theta(I_2 - I_1), I_2 - I_1\} \, \mathrm{d}z_1}{f(2, z|\theta)}, \\
&= \frac{1}{f(2, z|\theta)} \frac{1}{\sqrt{I_1}} \frac{1}{2\pi} \sqrt{\frac{I_2}{I_2 - I_1}} e^{-(z - \theta\sqrt{I_2})^2/2} \int_l^u z_1 \exp\left[-\frac{(z_1 - z\sqrt{I_1/I_2})^2}{2(I_2 - I_1)/I_2} \right] \mathrm{d}z_1,
\end{aligned}$$

where we have used Equation (3). To simplify the integral component of the above, note that by making the substitution $y = -(x-a)^2/b$, integration by parts gives

$$\int_l^u x \exp\left\{ -\frac{(x-a)^2}{b} \right\} \mathrm{d}x = \sqrt{\pi b} \left[\frac{b}{2}\{\phi(l, a, b/2) - \phi(u, a, b/2)\} + a\{\Phi(u, a, b/2) - \Phi(l, a, b/2)\} \right].$$

Applying this result with $a = z\sqrt{I_1/I_2}$ and $b = 2(I_2 - I_1)/I_2$, whilst also substituting in the form for $f(2, z|\theta)$ given in Equation (5), gives

$$\begin{aligned}
\hat{\theta}_{\text{UMVUE}}(2, z) &= \frac{I_2 - I_1}{I_2\sqrt{I_1}} \frac{\phi\{l_1, z\sqrt{I_1/I_2}, (I_2 - I_1)/I_2\} - \phi\{u_1, z\sqrt{I_1/I_2}, (I_2 - I_1)/I_2\}}{\Phi\{u_1, z\sqrt{I_1/I_2}, (I_2 - I_1)/I_2\} - \Phi\{l_1, z\sqrt{I_1/I_2}, (I_2 - I_1)/I_2\}} + \frac{z}{\sqrt{I_2}}, \\
&= \hat{\theta}_{\text{MLE}}(2, z) - \frac{I_2 - I_1}{I_2\sqrt{I_1}} \frac{\phi\{u_1, z\sqrt{I_1/I_2}, (I_2 - I_1)/I_2\} - \phi\{l_1, z\sqrt{I_1/I_2}, (I_2 - I_1)/I_2\}}{\Phi\{u_1, z\sqrt{I_1/I_2}, (I_2 - I_1)/I_2\} - \Phi\{l_1, z\sqrt{I_1/I_2}, (I_2 - I_1)/I_2\}},
\end{aligned}$$

as stated in the main manuscript.

Derivation of $\hat{\theta}_{\text{CUMVUE}}$

Note that the MLE based on the stage 2 data can be written as $(z\sqrt{I_2} - z_1\sqrt{I_1})/(I_2 - I_1)$. Then, moodifying the descriptions for $\hat{\theta}_{\text{UMVUE}}$, we have that

$$\begin{aligned}
\hat{\theta}_{\text{CUMVUE}}(2, z) &= \frac{\int_l^u \frac{z\sqrt{I_2} - z_1\sqrt{I_1}}{I_2 - I_1} \sqrt{I_2}\phi(u, \theta\sqrt{I_1}, 1)\phi\{z\sqrt{I_2} - z_1\sqrt{I_1}, \theta(I_2 - I_1), I_2 - I_1\} \, \mathrm{d}z_1}{f(2, z|\theta)}, \\
&= \frac{z\sqrt{I_2} - I_1\hat{\theta}_{\text{UMVUE}}(2, z)}{I_2 - I_1},
\end{aligned}$$

as required.

Matched pairs trial: Pocock boundaries

In Supplementary Figure 5, the performance of the nine estimators is shown for the matched pairs example. All parameters are as in Figure 6, except for l and u, which are modified to $u = -l = 2.178$ to correspond to Pocock stopping boundaries.

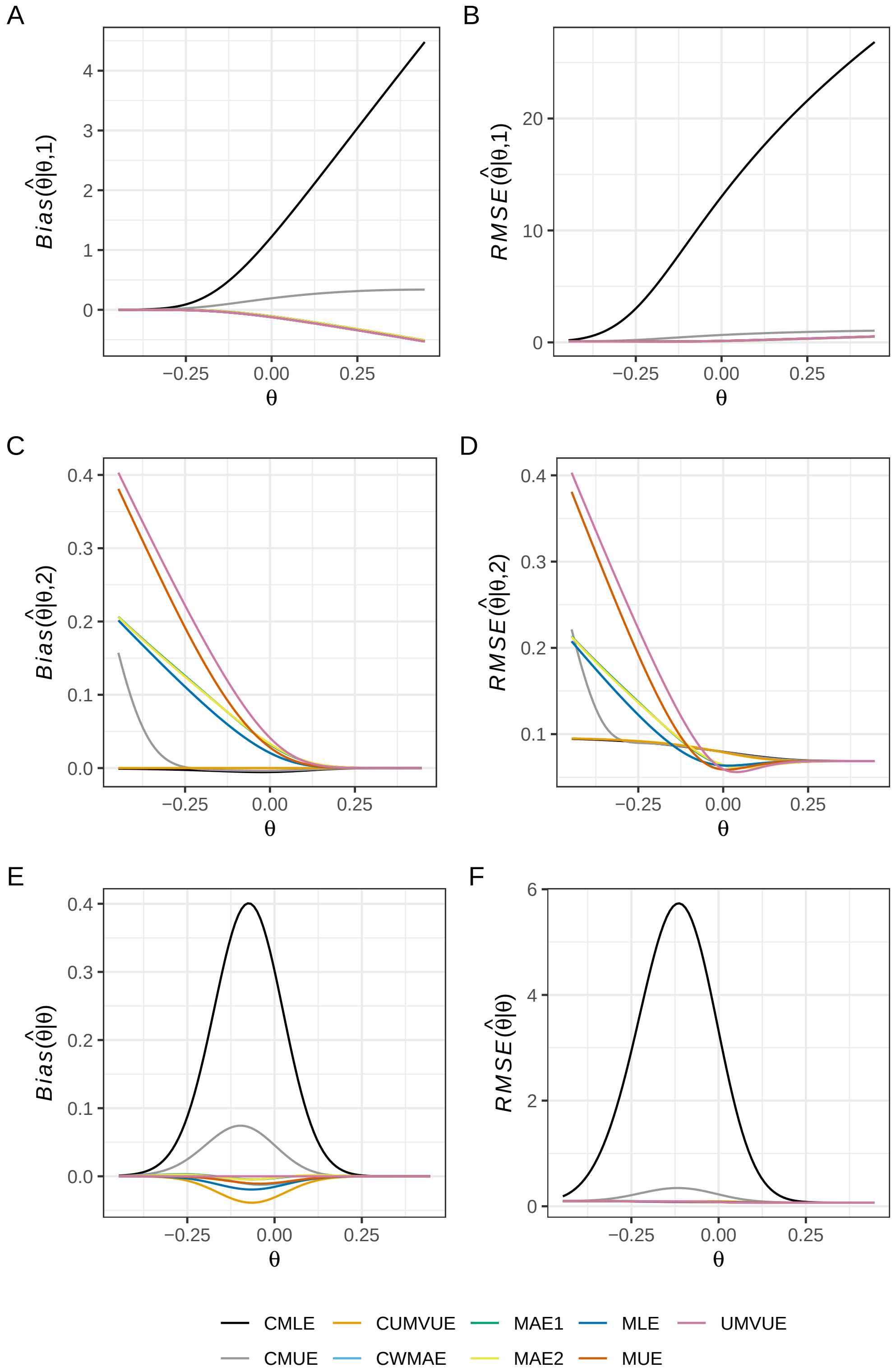

Supplementary Figure 1: The conditional and marginal bias and residual mean square error (RMSE) of the nine considered estimators is given for Example 1: two-arm survival data, with futility stopping only. Unlike in Figure 2, no restriction has been placed on the vertical axis limits.

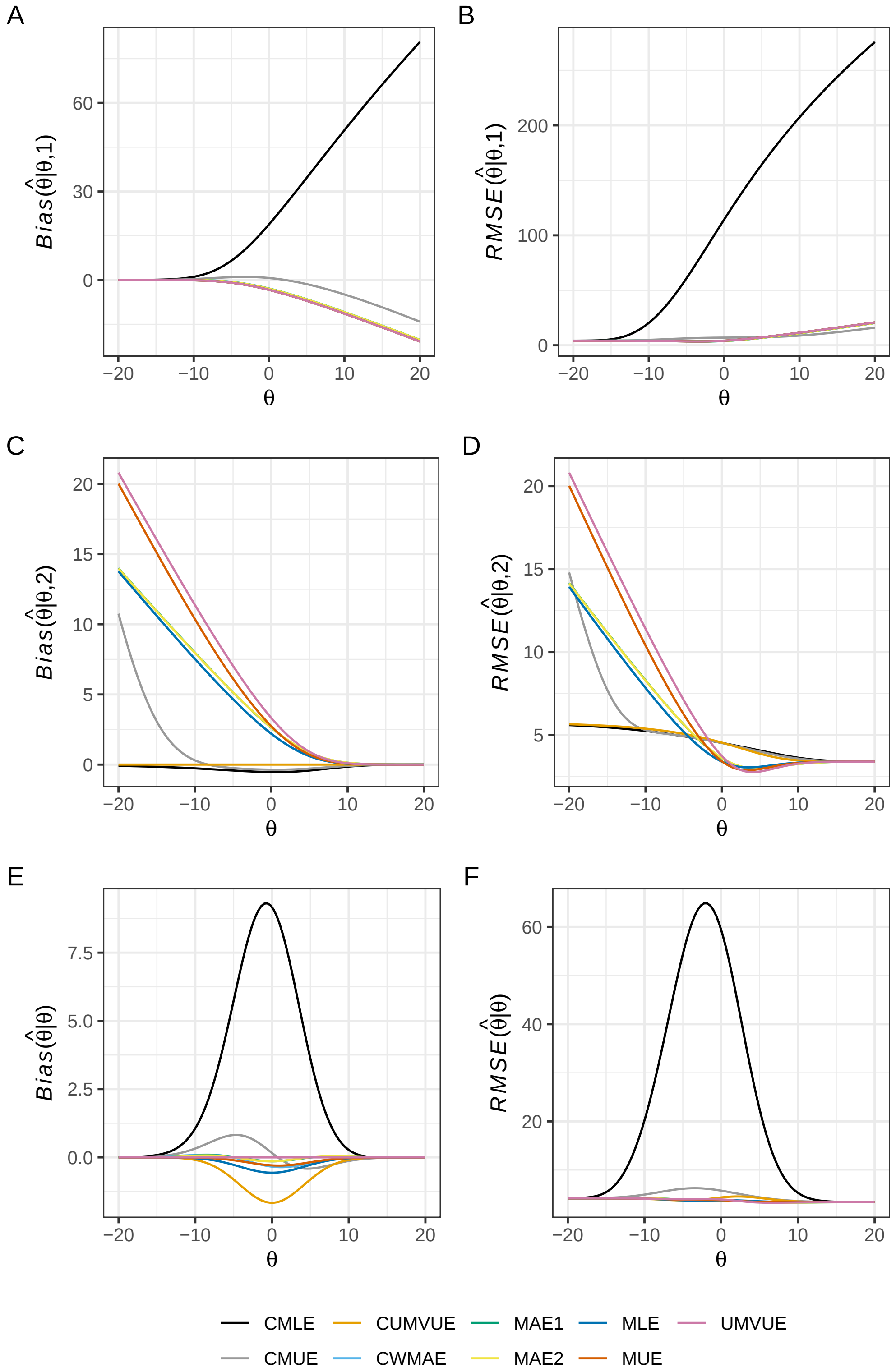

Supplementary Figure 2: The conditional and marginal bias and residual mean square error (RMSE) of the nine considered estimators is given for Example 2: single-arm normally distributed data. Unlike in Figure 3, no restriction has been placed on the vertical axis limits.

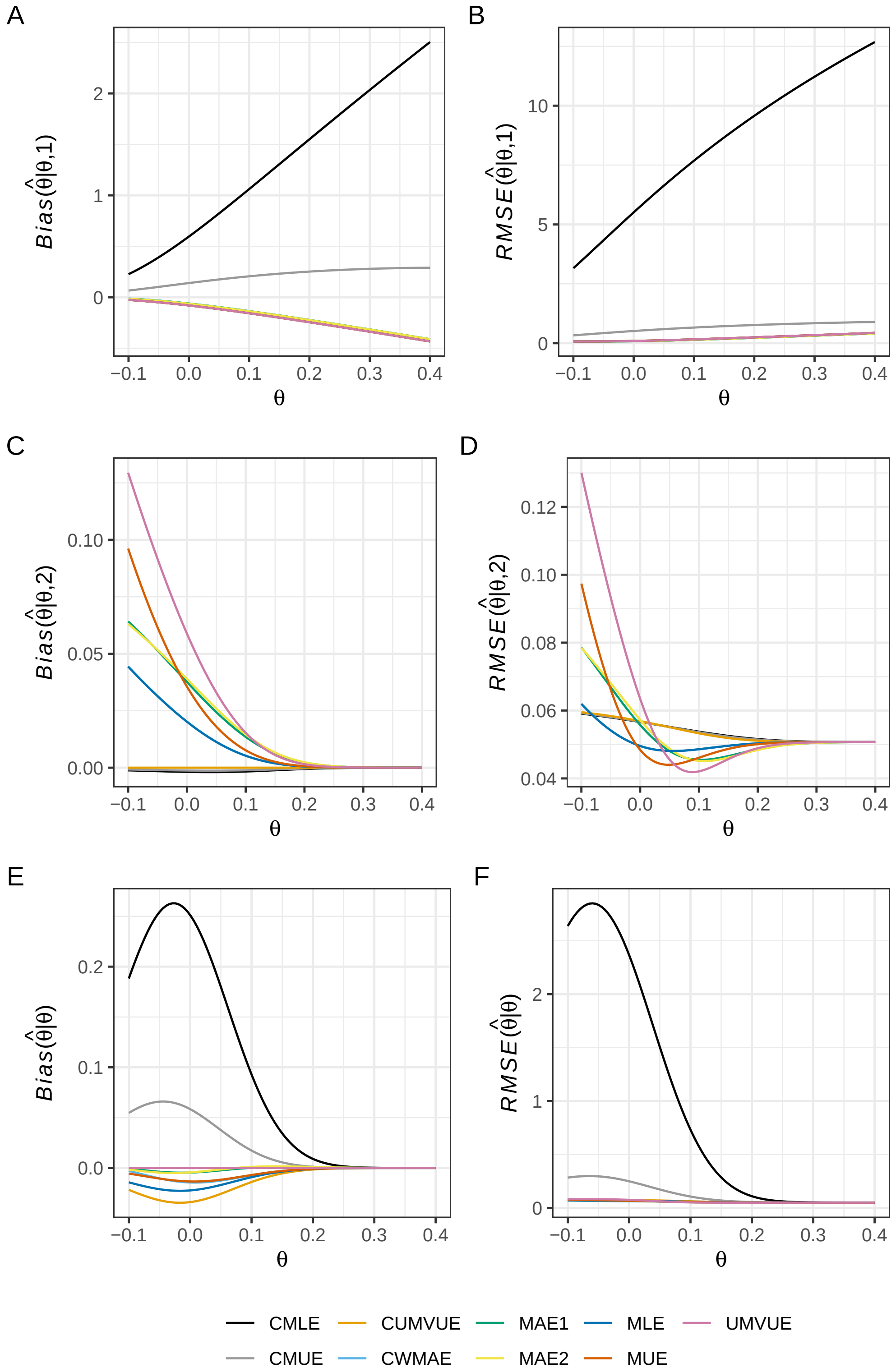

Supplementary Figure 3: The conditional and marginal bias and residual mean square error (RMSE) of the nine considered estimators is given for Example 4: single-arm Bernoulli distributed data. Unlike in Figure 4, no restriction has been placed on the vertical axis limits.

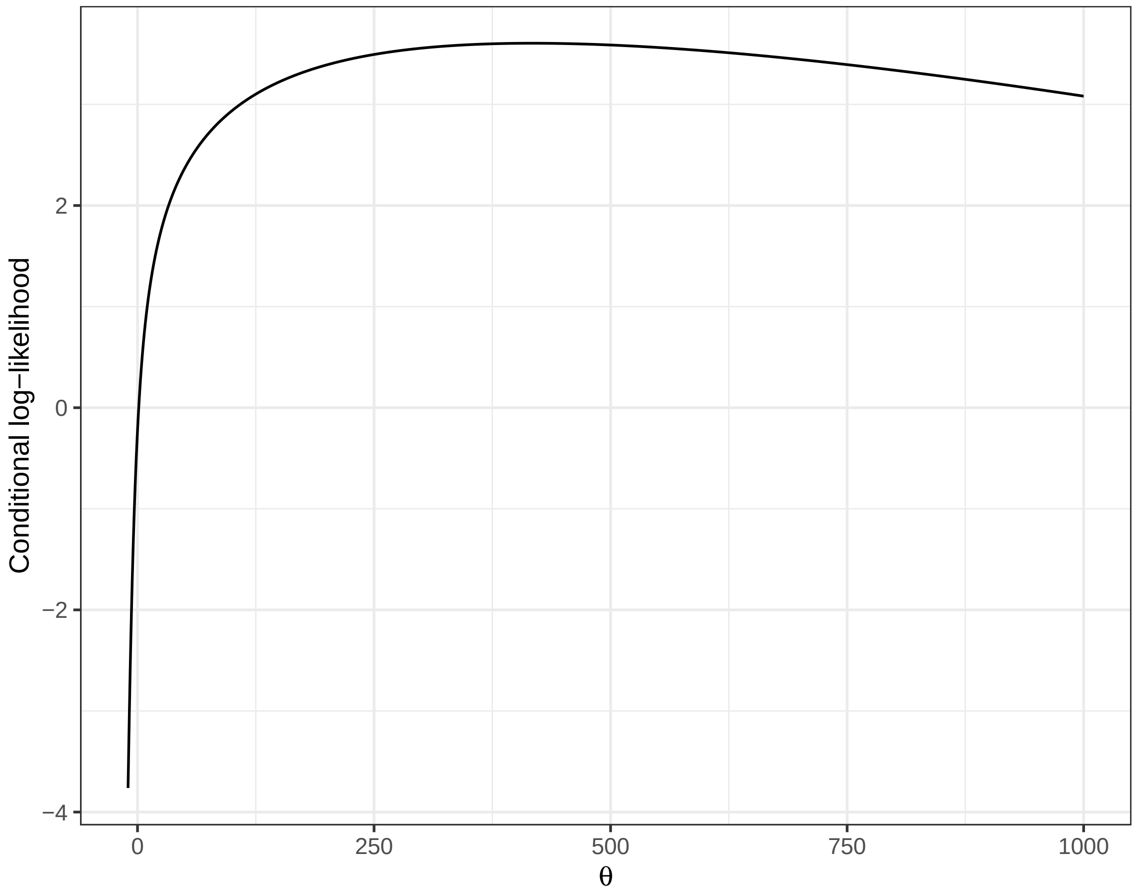

Supplementary Figure 4: Shows the form of the conditional log-likelihood function in the case that $k = 1$ and $z = -0.01$ in Example 2 ($l = 0$, $u = \infty$, $I_1 = 0.058$, $I_2 = 0.087$).

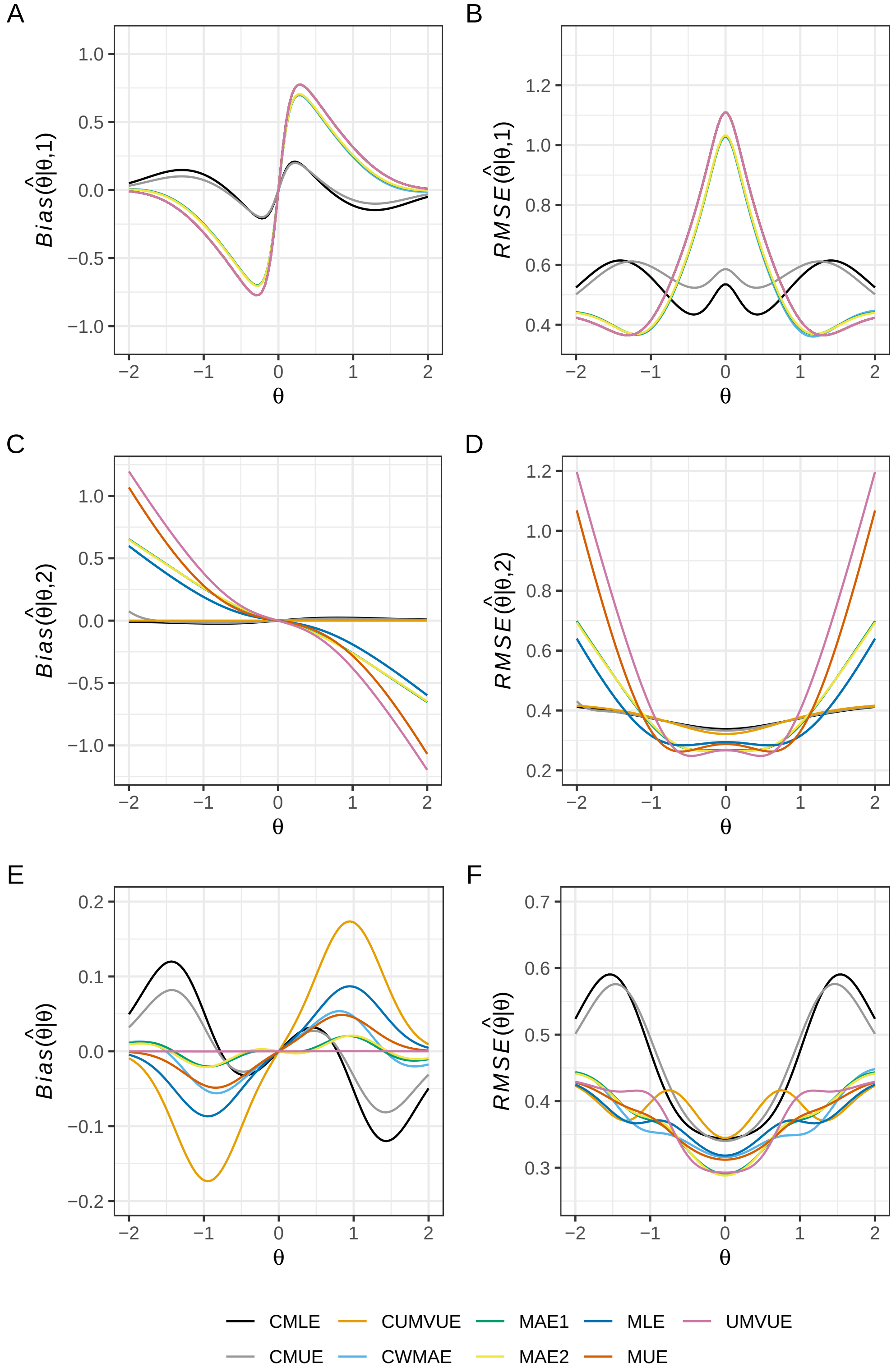

Supplementary Figure 5: The conditional and marginal bias and residual mean square error (RMSE) of the nine considered estimators is given for Example 5: matched pairs data, where the boundaries have been modified to be of Pocock form.
